# Integrated whole-genome gene expression analysis reveals an atlas of dynamic immune landscapes after myocardial infarction

**DOI:** 10.3389/fcvm.2023.1087721

**Published:** 2023-03-03

**Authors:** Yujue Wang, Yu Chen, Teng Zhang

**Affiliations:** ^1^Yueyang Hospital of Integrated Traditional Chinese and Western Medicine, Shanghai University of Traditional Chinese Medicine, Shanghai, China; ^2^Clinical Research Institute of Integrative Medicine, Shanghai Academy of Traditional Chinese Medicine, Shanghai, China; ^3^Laboratory of Clinical and Molecular Pharmacology, Yueyang Hospital of Integrated Traditional Chinese and Western Medicine, Shanghai University of Traditional Chinese Medicine, Shanghai, China

**Keywords:** myocardial infarction, heart failure, peripheral blood mononuclear cells, inflammation, integrated whole-genome gene expression analysis

## Abstract

**Introduction:**

Myocardial infarction (MI) is a deadly medical condition leading to irreversible damage to the inflicted cardiac tissue. Elevated inflammatory response marks the severity of MI and is associated with the development of heart failure (HF), a long-term adverse outcome of MI. However, the efficacy of anti-inflammatory therapies for MI remains controversial. Deciphering the dynamic transcriptional signatures in peripheral blood mononuclear cells (PBMCs) is a viable and translatable route to better understand post-MI inflammation, which may help guide post-MI anti-inflammatory treatments.

**Methods:**

In this work, integrated whole-genome gene expression analysis was performed to explore dynamic immune landscapes associated with MI.

**Results:**

GSEA and GSVA showed that pathways involved in the inflammatory response and metabolic reprogramming were significantly enriched in PBMCs from MI patients. Based on leukocyte profiles generated by xCell algorithm, the relative abundance of monocytes and neutrophils was significantly increased in PBMCs from MI patients and had positive correlations with typical inflammation-associated transcripts. Mfuzz clustering revealed temporal gene expression profiles of PBMCs during the 6-month post-MI follow-up. Analysis of DEGs and gene sets indicated that PBMCs from HF group were characterized by elevated and lasting expression of genes implicated in inflammation and coagulation. Consensus clustering generated 4 metabolic subtypes of PBMCs with molecular heterogeneity in HF patients.

**Discussion:**

In summary, integrated whole-genome gene expression analysis here outlines a transcriptomic framework that may improve the understanding of dynamic signatures present in PBMCs, as well as the heterogeneity of PBMCs in MI patients with or without long-term clinical outcome of HF. Moreover, the work here uncovers the diversity and heterogeneity of PBMCs from HF patients, providing novel bioinformatic evidence supporting the mechanistic implications of metabolic reprogramming and mitochondrial dysfunction in the post-MI inflammation and HF. Therefore, our work here supports the notion that individualized anti-inflammatory therapies are needed to improve the clinical management of post-MI patients.

## 1. Introduction

Myocardial infarction (MI) is a leading cause of cardiovascular morbidity and mortality worldwide (1). Existing therapies for MI, for instance, surgical revascularization as well as anti-platelet and anti-coagulation treatments, generally aim to resolve and ameliorate myocardial ischemia. Although these therapies have reduced the mortality rate of MI, a sizeable proportion of patients still develop pathological ventricular remodeling and progress to heart failure (HF) over time (2–4). HF, as an end-point condition of many cardiovascular diseases including MI, is plagued with notoriously high rates of hospitalization/rehospitalization and mortality (5, 6). Understanding of the mechanisms linking MI to the post-MI development of HF is important for timely prevention and control of HF.

The pathogenesis of post-MI HF is complex and remains incompletely understood. Inflammation plays a pivotal role in the pathogenesis of reperfusion injury and adverse ventricular remodeling following MI (7, 8), emerging as a critical player in the pathogenesis of HF (9). During MI, cellular debris and alarmins released from necrotic cardiomyocytes stimulate the immune cascade, triggering a sterile inflammatory response orchestrated through the activation and recruitment of immune cells into the infarcted myocardium (10, 11), local reprogramming of immune cells (11, 12), interplay of different immune cells as well as contributions from pericardial adipose tissue-specific source of immune cells (13). While the early inflammatory response after MI is required for initiating tissue repair (12), excessively heightened and prolonged inflammation leads to microvascular dysfunction and adverse ventricular remodeling, promoting the development of post-MI HF (14). Preclinical studies have demonstrated that genetic deletion or pharmacological inhibition of inflammatory signaling could attenuate inflammation and improve cardiac function (15, 16). Therefore, anti-inflammatory treatments have been valued as novel therapeutic strategies for the clinical management of MI patients (14, 17).

Although findings from the Canakinumab Anti-inflammatory Thrombosis Outcome Study (18) and Colchicine Cardiovascular Outcomes Trial (19) support the beneficial impact of post-MI anti-inflammatory treatments, clinical applications of the anti-inflammatory therapy are faced with challenges. One of the major challenges is to determine which patients should receive adjunctive anti-inflammatory therapies. Equally important, given that inflammatory mediators are pleiotropic and tightly controlled post-MI immune response is essential for initiating cardiac repair (14), the optimal timing to administer the anti-inflammatory therapy is critical. Extensive research has been carried out to examine gene expression dynamics in MI patients and model systems (16, 20, 21). However, few previous studies have yet been carried out to integrate multiple datasets to characterize the immune landscape of the clinically accessible peripheral blood mononuclear cells (PBMCs) from MI patients as well as to assess the heterogeneity of PBMCs with respect to the implication of inflammation in the development of post-MI HF.

Therefore, the current study primarily investigated the inflammatory activation of the post-MI PBMCs by gene set enrichment analysis (GSEA), gene set variation analysis (GSVA), and xCell algorithm. The heterogeneous and dynamic expression patterns of the post-MI PBMCs were analyzed using consensus clustering and Mfuzz soft clustering. Differences of PBMCs profiles between MI patient groups with and without HF were compared using GSEA. In addition, consensus clustering was employed to stratify HF PBMCs.

## 2. Materials and methods

### 2.1. Data collection and preprocessing

Microarray datasets were obtained from the publicly available Gene Expression Omnibus (GEO) database.^1^ Eleven datasets were included and analyzed in this study (22–28) (Table 1). The gene expression matrix derived from GSE59867 and GSE62646 was merged and normalized. The *ComBat* function of the *sva* package was applied to eliminate batch effects (29). Distribution and variation of the samples before and after batch normalization were evaluated by principal component analysis (PCA). The normalized gene expression matrix was used for subsequent analyses. Each of the remaining data set was processed independently.

**Table 1:** Summary of the datasets analyzed in the present study.

Accession	Sample source	Organism	Sample size	Group	Platform
GSE59876	PBMCs	*H. sapiens*	436	46 SCAD + 390 STEMI^1^	GPL62646
GSE62646	PBMCs	*H. sapiens*	98	14 SCAD + 84 STEMI^2^	GPL62646
GSE123342	Whole blood	*H. sapiens*	98	22 SCAD + 67 AMI	GPL17586
GSE48060	Whole blood	*H. sapiens*	52	21 NC + 31 AMI	GPL570
GSE141512	PBMCs	*H. sapiens*	12	6 NC + 6 MI	GPL17586
GSE19339	Thrombus-derived leukocytes	*H. sapiens*	8	4 NC + 4 ACS	GPL570
GSE66360	CECs	*H. sapiens*	99	50 NC + 49 AMI	GPL570
GSE109048	Platelets	*H. sapiens*	38	19 SCAD + 19 STEMI	GPL17586
GSE159657	Exosomes	*H. sapiens*	18	8 SCAD + 10 ACS	GPL24676
GSE77343	PBMCs	*H. sapiens*	197	197 HF	GPL11532
GSE126446	Platelets	*H. sapiens*	8	4 MPs + 4 RPs	GPL18573

1 Grouping of the samples in GSE59867: SCAD (*n* = 46), MI at admission (*n* = 111), MI at discharge (*n* = 101), 1 month after MI (*n* = 95) and 6 months after MI (*n* = 83).

2 Grouping of the samples in GSE62646: SCAD (*n* = 14), MI at admission (*n* = 28), MI at discharge (*n* = 28) and 6 months after MI (*n* = 28).

ACS, acute coronary syndrome; AMI, acute myocardial infarction; CEC, circulating endothelial cells; HF, heart failure; MI, myocardial infarction; MPs, mature platelets; NC, normal control; PBMCs, peripheral blood mononuclear cells; RPs, reticulated platelets; SCAD, stable coronary artery disease; STEMI, ST-elevation myocardial infarction.

### 2.2. Screening of differentially expressed genes

DEGs were screened by the *Limma* package (30). DEGs analyses were performed with a *p* < 0.05 and |log_2_FC| > 0.585 (|FC| > 1.5) signifying statistical significance. Volcano plots for visualization were drawn using the *ggplot2* package.

### 2.3. Gene set enrichment analysis

Gene set enrichment analysis was employed to identify enriched gene sets and the leading-edge gene subsets which contributed most to the enriched pathways. GSEA was performed using the *clusterProfiler* R package (31). Human genes were annotated using *org.Hs.eg.db* package. Gene Ontology (GO) terms^2^ and Kyoto Encyclopedia of Genes and Genomes (KEGG) pathways^3^ were used as the reference gene sets. Gene sets with an absolute normalized enrichment score (|NES|) > 1, *p* value <0.05 and adjusted *p* value (*p*.adj) < 0.25 were considered to be significantly enriched (32). The Benjamini–Hochberg procedure was used to control the false discovery rate. Visualization was performed using the *ggplot2* package.

### 2.4. Gene set variation analysis

Gene set variation analysis was performed to quantify pathway activities of the samples using *GSVA* R package (33). The Hallmark gene sets from MSigDB (34) were selected as the reference gene sets. *Limma* package was used to define the differentially expressed gene sets. A *p* value <0.05 was set as the cut-off criterion.

### 2.5. Leukocytes profiling analysis

We employed the xCell algorithms to estimate the relative abundance of leukocytes in PBMCs (35) using the online analytical platform.^4^ The Charoentong signatures were selected as the reference gene sets.

### 2.6. Time series analysis

Time series analysis was performed using *Mfuzz* (2.50.0) package (36). Functional enrichment analyses of gene clusters were generated by the online analytical platform METASCAPE.^5^ A *p* value *<*0.01 was taken as the threshold for statistical significance.

### 2.7. Consensus clustering

The consensus clustering was executed using *ConsensusClusterPlus* package (37). To ensure the classification stability, the analyses was repeated 1,000 times with different initial conditions for modeling fitting to assure internal consistency (parameters: reps = 100, *p* Item = 0.8, *p* Feature = 1). Each sample of PBMCs from patients with stable coronary artery disease (SCAD) and MI patients was assigned to either one of two sub-clusters, based on the expression levels of genes belonging to the MSigDB (34) gene sets “GOBP_INFLAMMATORY_RESPONSE” (*n* = 773). Ward.D and Euclidean distances were used as the clustering algorithm and distance metric, respectively. PBMCs from HF patients were classified into three sub-clusters based on our custom gene sets containing 26 glycolytic genes and 47 oxidative phosphorylation (OXPHOS) genes (Supplementary Data Sheet 1). Ward.D and Spearman distances were used as the clustering algorithm and distance metric, respectively.

### 2.8. Correlation analysis

Correlation analysis was performed to evaluate correlations between the abundance of and the expression level of pro-inflammatory transcripts in leukocytes. The correlation coefficient was obtained by performing the Spearman’s correlation analysis. Visualization was performed using the *ggplot2* package.

### 2.9. Statistical analyses

Statistical analyses and plotting were performed using R or GraphPad Prism 9 software. Given that the gene expression data do not fit a normal distribution by the Shapiro–Wilk test, two-group comparisons were determined using the Mann–Whitney non-parametric test. Multiple-group comparisons were determined using Kruskal–Wallis test with Dunn’s correction. Comparison of paired samples was performed by using Wilcoxon matched-pairs test. A spectrum of gene expressions was visualized by Box-Whisker plots or violin plots. The Box-Whisker plots denoted median + quartiles (box) and range (whiskers) and the violin plot showed the density distribution of all data points. For all tests applied, statistical differences were determined significant at *p* < 0.05.

## 3. Results

### 3.1. Activation of pro-inflammatory and glycolytic pathways in PBMCs is a general feature of MI patients

**Figure 1:**
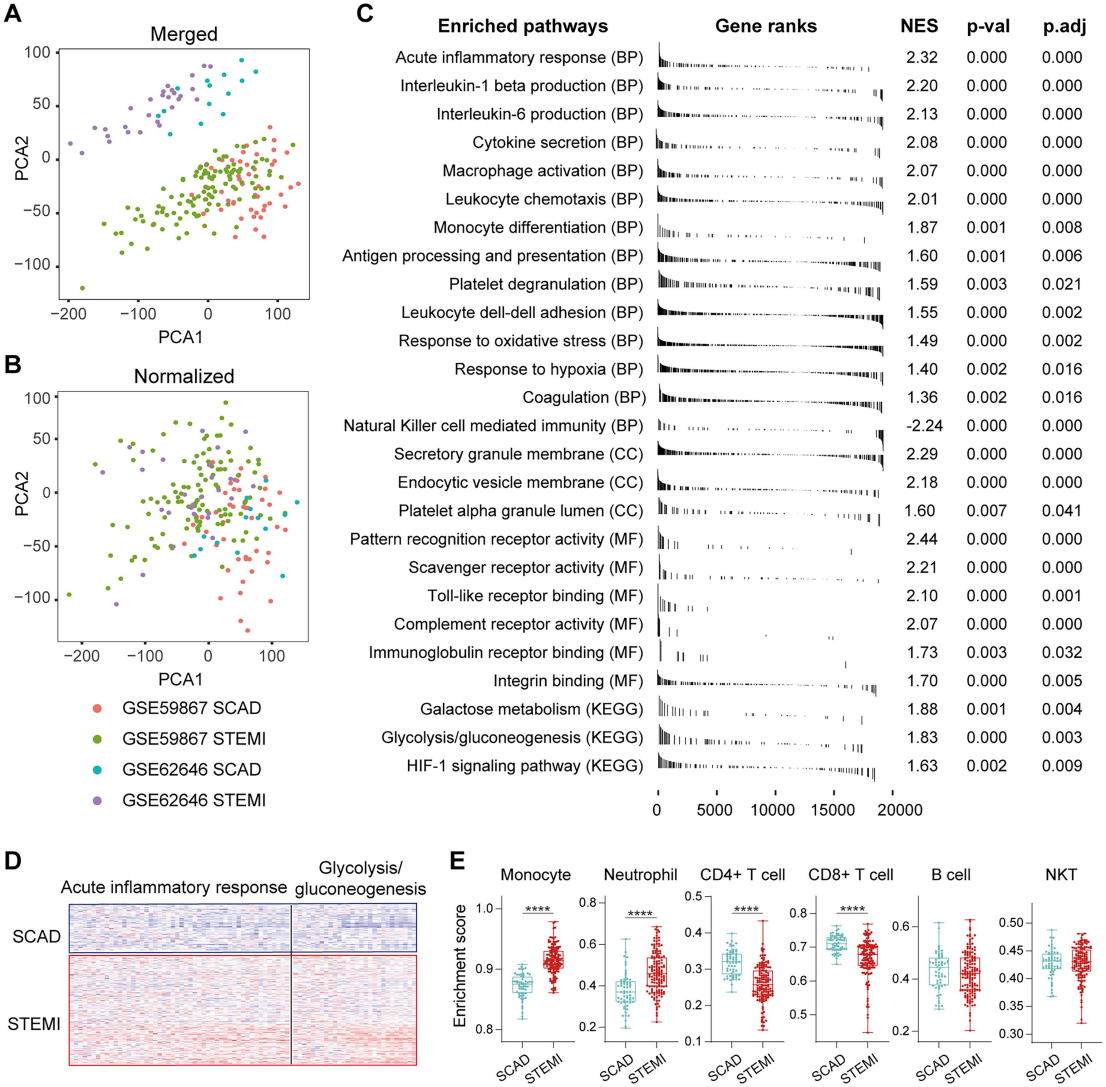
Activation of pro-inflammatory and glycolytic pathways in PBMCs from the MI patients. Two-dimensional PCA plots were used to visualize the distribution and variation of samples before **(A)** and after **(B)** batch normalization (*n* = 199). Each dot represented one sample and was colored by group. **(C)** Representative gene sets enriched in PBMCs from the MI patients at admission. Each vertical bar represented one gene, with the location of the bar indicating the occurrence of that gene in the gene list, and the height of the bar representing the relative fold change. Gene sets with a |NES| > 1, *p* value<0.05, *p*.adj < 0.25 were considered to be significantly enriched. **(D)** Heatmap depicted the expression of the leading-edge genes contributed most to the indicated pathways. **(E)** The relative abundance of leukocytes in PBMCs was computed based on xCell algorithm. Two-group comparisons were made using the Mann–Whitney non-parametric test with *p* value <0.05 signifying statistical significance. The Box-Whisker plots denoted median + quartiles (box) and range (whiskers). *p < 0.05, **p < 0.01, ***p < 0.001, ****p < 0.0001. BP, biological process; CC, cell component; KEGG, Kyoto Encyclopedia of Genes and Genomes; MF, molecular function; NES, normalized enrichment score; PCA, principal component analysis; *p*.adj, adjusted *p* value; *p*-val, *p* value; SCAD, stable coronary artery disease; STEMI, ST-elevation myocardial infarction.

To better understand the MI-associated inflammatory processes, we began with exploring alterations of gene expression profiles associated with the MI PBMCs. Microarray datasets of GSE59867 and GSE62646 were obtained from the publicly available GEO database. In the cohort of GSE59867, MI patients at admission (*n* = 111) who were indicated for percutaneous coronary interventions (PCI) were assigned as the study group. Age-, gender-, and BMI-matched SCAD patients (*n* = 46) without a history of MI were used as the reference group. The baseline demographic and clinical characteristics of the two groups were included in the original publication (17). In the cohort of GSE62646, MI patients at admission (*n* = 28) who were indicated for PCI were assigned as the study group. Gender- and BMI-matched SCAD patients (*n* = 14) without a history of MI were used as the reference group. It was noted that the patients in the SCAD group were older than the patients in the MI group in this cohort (67.8 years of age in the SCAD control group vs. 55.4 years of age in the MI group). Given that age is a non-modifiable risk factor for chronic inflammation associated with age-associated diseases (38), it is possible that the difference in age here might not cause overestimation of the potential association of inflammation with MI. The baseline demographic and clinical characteristics of the two groups were showed in the original publication (23). Given that both GSE59867 cohort and GSE62646 cohort were carried out with similar designs and the PBMCs samples from these two experiments were processed on the same platform (GPL6244), we merged and normalized the two data sets to expand the sample size and to increase the reliability of the analysis. The combined dataset contained 139 PBMCs samples from the MI patients at admission and 60 PBMCs samples from the SCAD patients. Sample distribution without (Figure 1A) and with (Figure 1B) batch-normalization adjustment was visualized using PCA plots. The normalized gene expression matrix was used for subsequent analyses. The alterations of gene expression profiles associated with the MI PBMCs were examined with PBMCs from SCAD patients serving as the control. GSEA revealed that inflammatory response (e.g., cytokine secretion, leukocyte chemotaxis and leukocyte cell–cell adhesion, etc.) and metabolic pathways (e.g., galactose metabolism and glycolysis/gluconeogenesis, etc.) were significantly enriched in the MI group (Figure 1C). Furthermore, the expression patterns of the core leading-edge genes contributed most to glycolysis were similar with that of acute inflammatory response (Figure 1D). Interestingly, several pathways involved in platelet activation, for instance, platelet degranulation, coagulation, and platelet alpha granule lumen, were significantly enriched (Figure 1C). Enrichment of platelet-associated pathways in the MI PBMCs may in part result from increased leukocyte-platelet aggregates and coagulation abnormalities (39, 40). Detailed GSEA enrichment results can be found in Supplementary Data Sheet 2. Moreover, the xCell analysis revealed that in the PBMCs derived from MI patients, the relative abundance of monocytes and neutrophils was significantly increased and the relative abundance of CD4^+^ lymphocytes and CD8^+^ lymphocytes was significantly decreased (Figure 1E), consistent with that reported in the literature (41). This increase in blood neutrophils and monocytes might result from exodus of neutrophils and monocytes from the hematopoietic stem and progenitor cells in the bone marrow (16). It was noted that increased abundance of neutrophils could in part be caused by the presence of low-density granulocytes (LDG) in the MI PBMCs (42). Taken together, the results here indicate that PBMCs from the MI patients at admission are molecularly marked by pro-inflammatory and glycolytic gene signatures.

### 3.2. The MI patients are heterogeneous with respect to the pro-inflammatory activation of PBMCs

**Figure 2:**
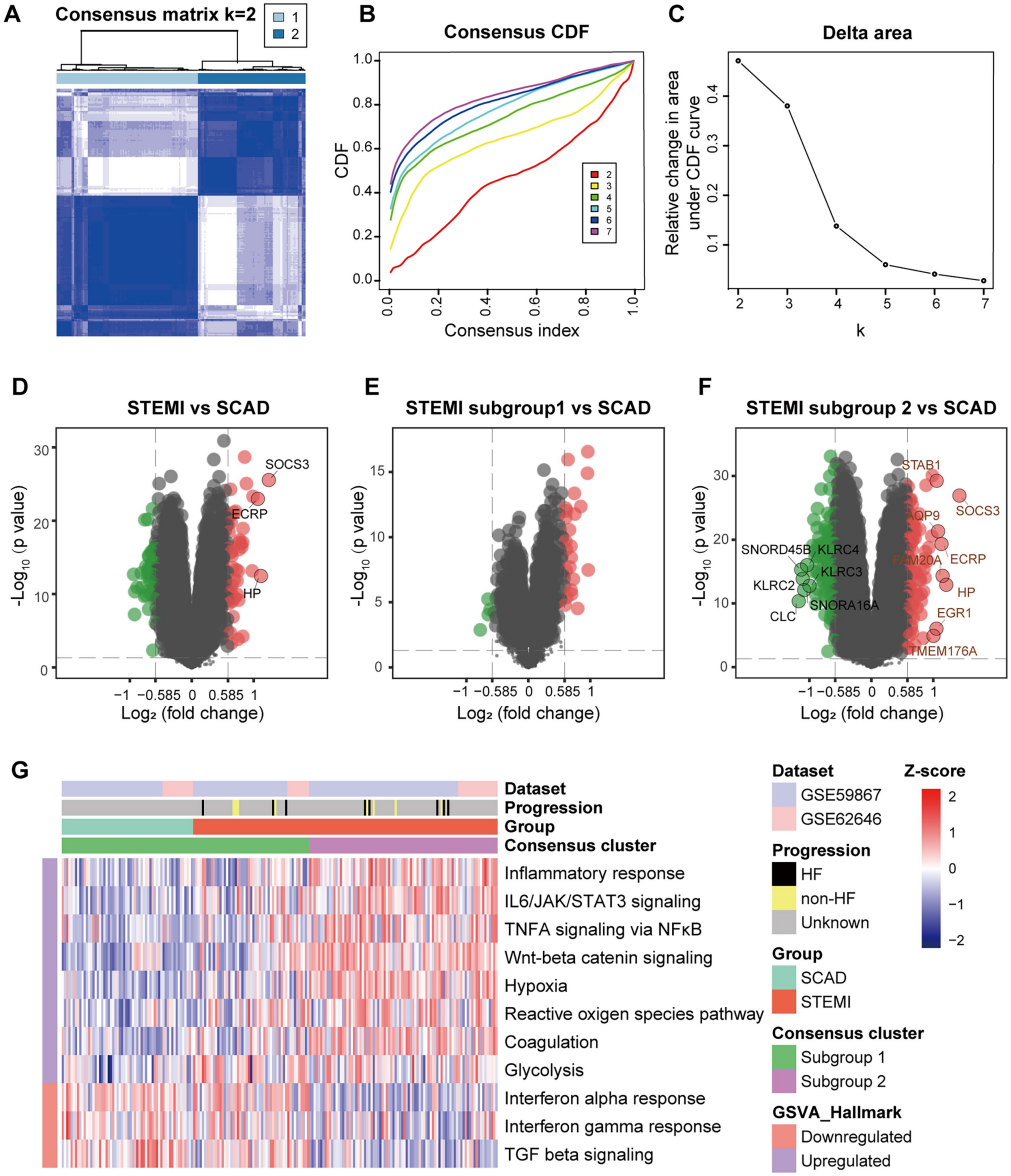
Stratification of the MI PBMCs based on inflammatory response. **(A)** A two-cluster solution of PBMCs yielded by consensus clustering (*n* = 199). **(B)** CDF plot depicted the cumulative distribution functions of the consensus matrix for *k* = 2–7. **(C)** Delta area plot depicted the relative change in area under CDF curve for *k* = 2–7. **(D)** Volcano plots showed the DEGs when STEMI was compared to CAD. **(E)** Volcano plots showed the DEGs when STEMI subgroup 1 was compared to CAD. **(F)** Volcano plots showed the DEGs when STEMI subgroup 2 was compared to CAD. The DEGs with an adjusted *p* value<0.05 and |log2FC| > 1 were circled and labeled. **(G)** Heatmap visualized the pathway activities of PBMCs quantified by GSVA. The color scale reflected Z-scores with red indicating relatively high expression and blue relatively low expression. CDF, cumulative distribution functions; DEGs, differential expressed genes; GSVA, gene set variation analysis; HF, heart failure; SCAD, stable coronary artery disease; STEMI, ST-elevation myocardial infarction.

Consensus clustering was further performed to clarify if augmented inflammation is universally or selectively associated with the MI patients. According to the expression of genes belonging to the MSigDB gene sets “Inflammatory response” (*n* = 773), each sample was assigned to either one of the two subtypes using consensus clustering (Figure 2A). Based on the expression of selected genes, *k* = 2 seemed to be an adequate selection with the clustering stability increasing from *k* = 2–7 (Figures 2B,C). Out of the 139 MI patients, 53 were classified into the subgroup 1 and 86 were classified into the subgroup 2. In contrast, all SCAD patients were classified into the subgroup 1, which was characterized by a hypo-inflammatory phenotype (Figures 2D–G). To further compare the phenotypic differences of PBMCs between the two subgroups, DEGs analyses were performed with a |log_2_FC| > 0.585 and a *p* value of <0.05 signifying statistical significance. GSVA was employed to quantify the hallmark pathway activities of the samples. Detailed GSVA results can be found in Supplementary Data Sheet 3. The results revealed that the expression profiles of MI PBMCs in the subgroup 1 shared more similarity to the profiles in SCAD PBMCs instead of resembling the profiles of the MI PBMCs in the subgroup 2 (Figures 2D–F). Multiple hallmark pro-inflammatory pathway activities were elevated in the MI group, particularly in the subgroup 2, including inflammatory response, IL6/JAK/STAT3 signaling, TNFA signaling *via* NFκB, Wnt-beta catenin signaling, hypoxia and glycolysis (Figure 2G). These results indicate a portion of the MI patients are classified into the hyper-inflammatory group, while the remaining patients display less-severe inflammation. Furthermore, our analysis here suggests that consensus clustering could be a feasible approach to classify MI patients into the hyper-inflammatory subtype and the hypo-inflammatory subtype.

### 3.3. MI is characterized by a pan increase in pro-inflammatory transcripts in the circulation

**Figure 3:**
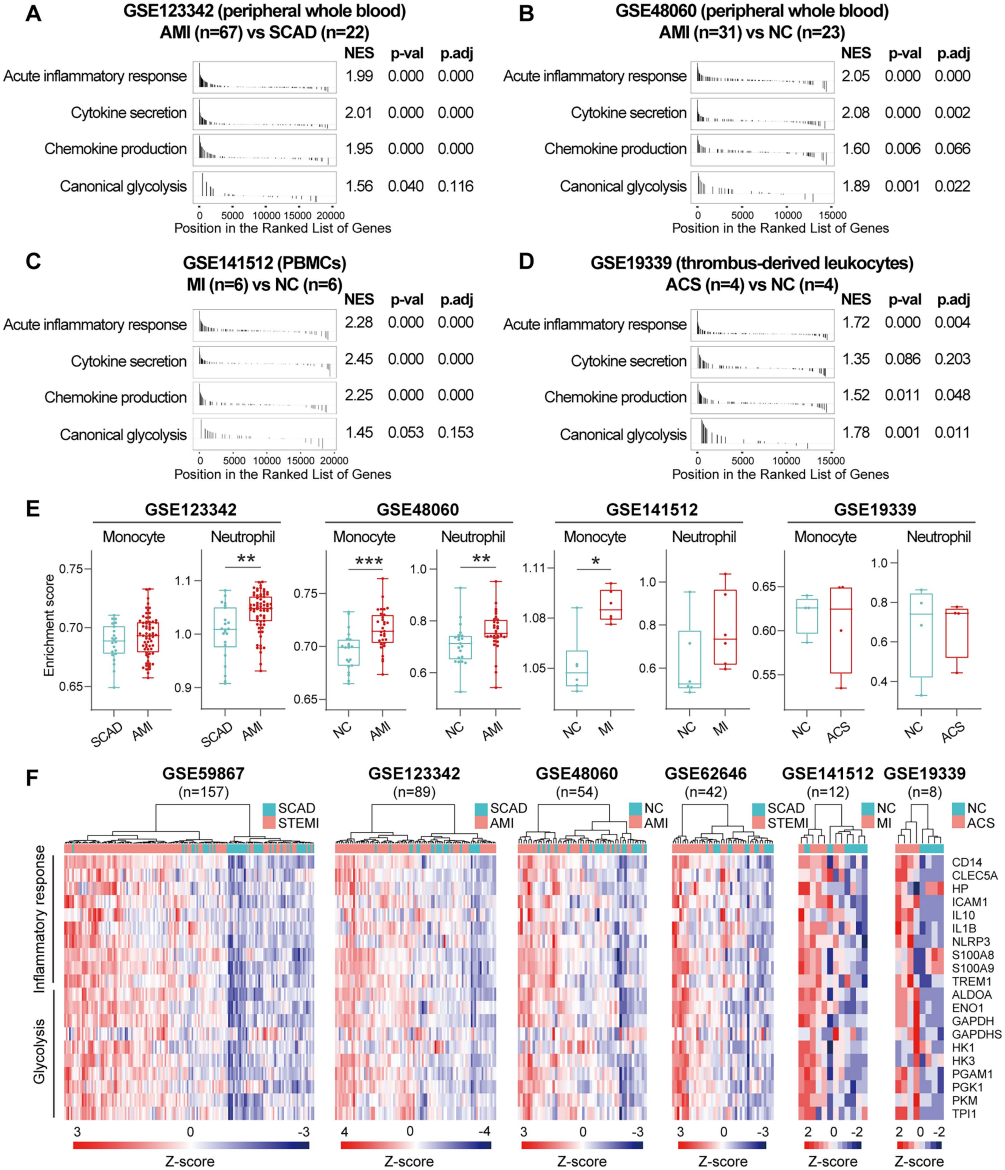
Increased expression of pro-inflammatory and glycolytic genes in PBMCs and peripheral whole blood from the MI patients. **(A–D)** GSEA plots showed a significant enrichment of gene sets involved in inflammatory response and glycolysis in the MI patients. Gene sets with *p* value<0.05, *p*.adj < 0.25 and |NES| > 1 were considered to be significantly enriched. **(E)** The Box-Whisker plots showed the relative abundance of monocytes and neutrophils in PBMCs. The central line represented the median value and the box bounds represented the interquartile range; whiskers represented min to max. Two-group comparisons were determined using the Mann–Whitney non-parametric test, with *p* value<0.05 signifying statistical significance. **(F)** Heatmap depicted the expression of representative leading-edge genes contributed most to the enriched gene sets. *p < 0.05, **p < 0.01. ACS, acute coronary syndrome; AMI, acute myocardial infarction; MI, myocardial infarction; NC, normal control; NES, normalized enrichment score; PBMCs, peripheral blood mononuclear cells; *p*.adj, adjusted *p* value; *p*-val, *p* value; SCAD, stable coronary artery disease; STEMI, ST-elevation myocardial infarction.

**Figure 4:**
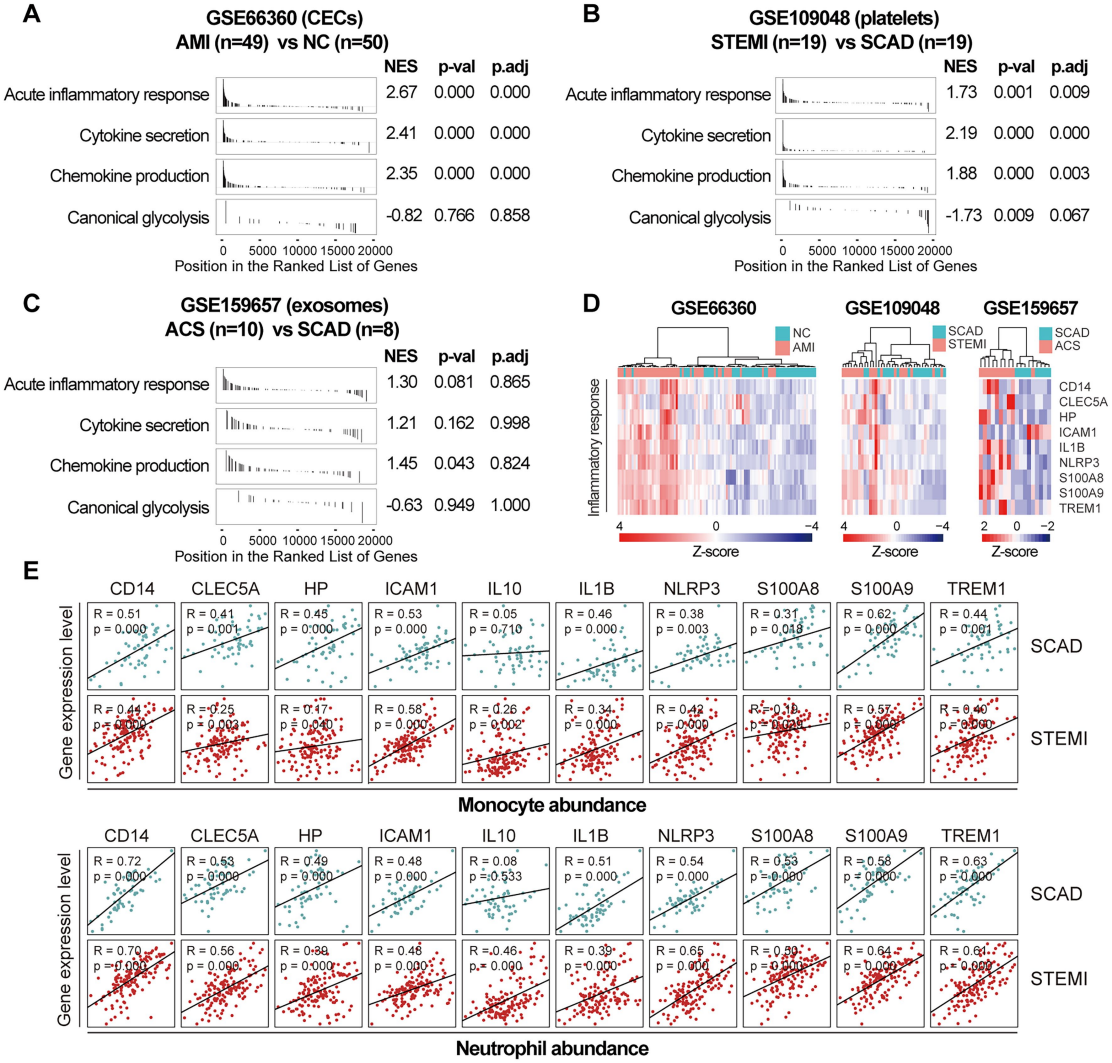
Increased expression of pro-inflammatory genes in CECs, platelets and exosomes from the MI patients. GSEA plots showed a significant enrichment of gene sets involved in inflammatory response in CECs **(A)** and platelets **(B)** from the MI patients. Gene sets with *p* value<0.05, *p*.adj < 0.25 and |NES| > 1 were considered to be significantly enriched. **(C)** GSEA plots of the exosomal gene sets. **(D)** Heatmap depicted the expression of the representative pro-inflammatory genes. **(E)** Scatter plots showed the correlations between the abundance of monocytes or neutrophils and the expression level of the representative pro-inflammatory genes in both SCAD patients and MI patients, using the combined and normalized dataset of GSE59867 and GSE62646. Statistical analysis was performed using Spearman’s correlation test. Correlation coefficient and *p* value were noted in the top left corner of each plot. ACS, acute coronary syndrome; AMI, acute myocardial infarction; CECs, circulating endothelial cells; LAD, left anterior descending; NC, normal control; NES, normalized enrichment score; *p*.adj, adjusted *p* value; *p*-val, *p* value; SCAD, stable coronary artery disease; STEMI, ST-elevation myocardial infarction.

Next, we sought to validate the foregoing observations and evaluate changes in inflammatory markers by analyzing GSE123342, GSE48060, GSE141512, GSE19339, GSE66360, GSE109048, and GSE159657 data sets from additional MI cohorts. For each data set, GSEA was performed by ranking the gene sets after differential expression analysis. Detailed GSEA enrichment results can be found in Supplementary Data Sheet 4. Hallmark pathways including acute inflammatory response, cytokine secretion, chemokine production and canonical glycolysis were selected to assess pro-inflammatory activation of the samples. GSEA revealed that gene sets associated with acute inflammatory response, cytokine secretion, chemokine production and canonical glycolysis were enriched in the whole blood (Figures 3A,B), PBMCs (Figure 3C), and thrombus-derived leukocytes (Figure 3D) from the MI patients. Based on the results from the xCell analysis, the increase in the relative abundance of monocytes in the post-MI blood was validated in GSE48060 and GSE141512 data sets and the increase in neutrophils was verified in GSE123342 and GSE48060 data sets (Figure 3E). Genes of interest included those that showed a significant upregulation in over two third of the indicated datasets. The representative leading-edge genes contributed most to the enriched gene sets involved in inflammatory response and glycolysis were listed in Figure 3F. The post-MI peripheral blood was generally marked by an upregulation of pro-inflammatory genes (e.g., CD14, CLEC5A, HP, ICAM1, IL10, IL1B, NLRP3, S100A8, S100A9, TREM1, etc.) and glycolytic genes (e.g., ALDOA, ENO1, GAPDH, GAPDHS, HK1, HK3, PGAM1, PGK1, PKM, TPI1, etc.). In addition, the circulating endothelial cells (CECs; Figure 4A) and platelets (Figure 4B) from the MI patients also manifested a significant enrichment of pro-inflammatory gene sets. Although these specific gene sets did not appear to be enriched in circulating exosomes (Figure 4C), the exosomal level of the classical inflammation markers such as CD14, CLEC5A, IL1B, NLRP3, S100A8, S100A9, and TREM1 was significantly upregulated (Figure 4D). Detailed GSEA enrichment results of these datasets can be found in Supplementary Data Sheet 4. The strength of the relationship between hallmark inflammatory markers and relative abundance of leukocytes was assessed using Spearman’s correlation coefficients. The results revealed that almost all of these pro-inflammatory transcripts exhibited positive correlations with the abundance of monocytes and neutrophils (Figure 4E), indicating monocytes and neutrophils could be key players in the MI-associated acute inflammatory responses. Our results from multiple validation datasets further confirm that MI patients are generally characterized by a phenotype of pro-inflammatory activation in the blood. Furthermore, the results here reveal that the robust post-MI upregulation of the hallmark pro-inflammatory transcripts is a common phenomenon in the peripheral whole blood, PBMCs, thrombus-derived leukocytes, CECs, platelets and exosomes.

The temporal evolution of gene expression profiles of PBMCs from the MI patients

**Figure 5:**
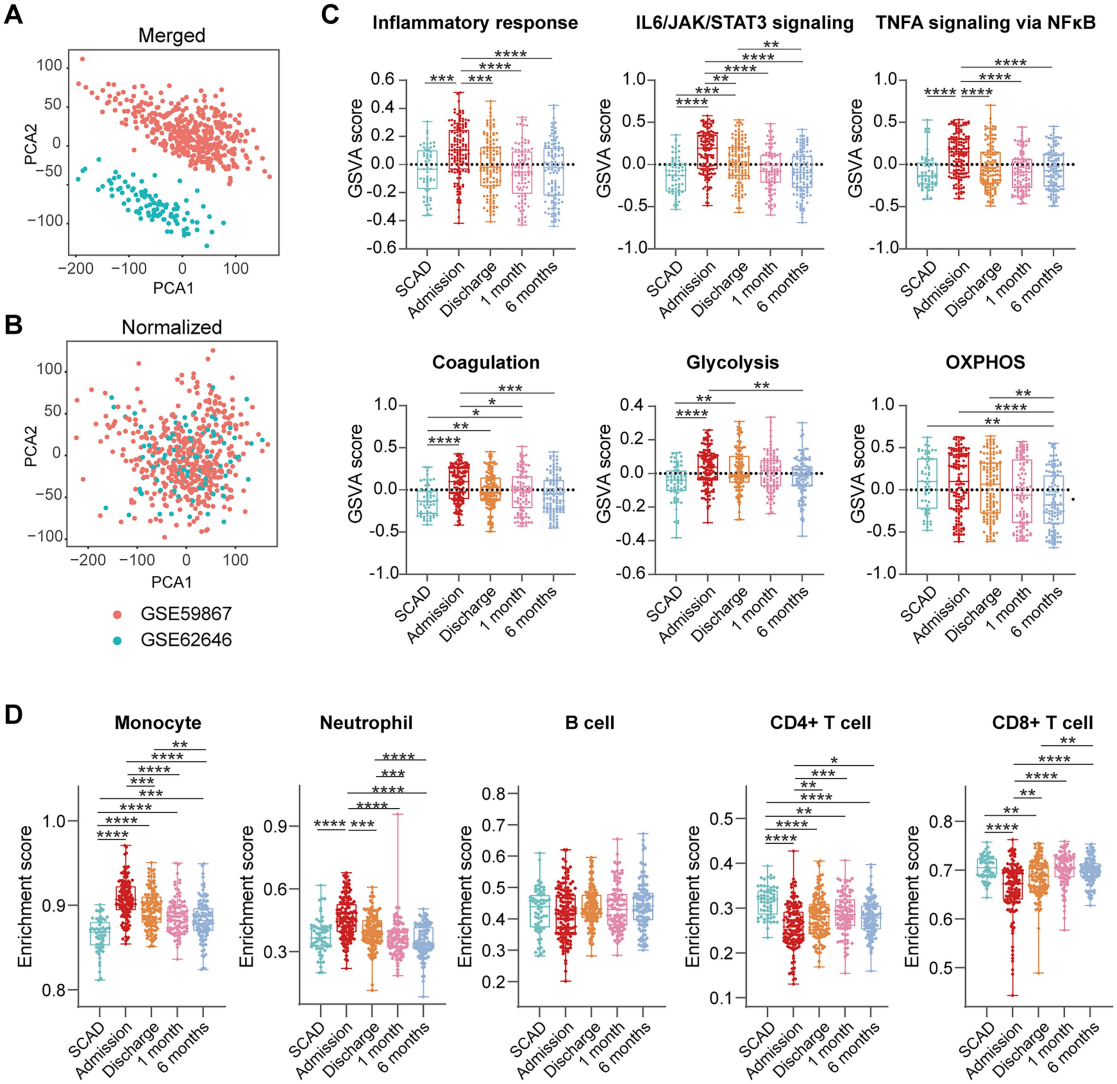
Temporal evolution of the post-MI PBMCs gene expression profiles assessed by GSVA and xCell. Two-dimensional PCA plots visualized the distribution and variation of samples before **(A)** and after **(B)** batch normalization (*n* = 534). Each dot in the plots represented one sample. **(C)** Boxplots depicted the pathway activities of PBMCs quantified by GSVA. **(D)** Boxplots depicted the abundance of the indicated cell components quantified by xCell. The Box-Whisker plots denoted median + quartiles (box) and range (whiskers). Multiple-group comparisons were made using Kruskal–Wallis test with Dunn’s correction. *p < 0.05, **p < 0.01, ***p < 0.001, ****p < 0.0001. OXPHOS, oxidative phosphorylation; SCAD, stable coronary artery disease.

**Figure 6:**
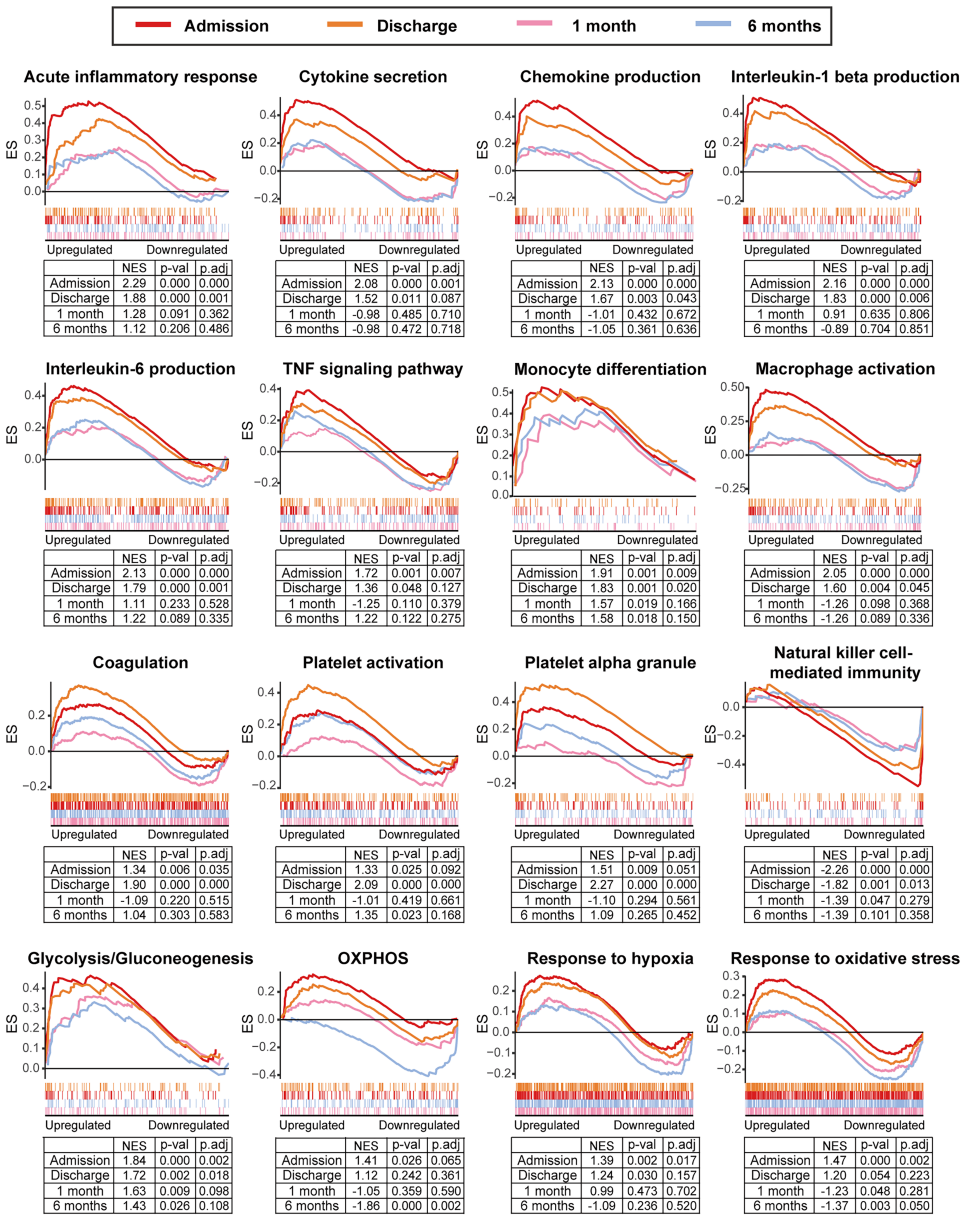
Temporal evolution of the post-MI PBMCs gene expression profiles assessed by GSEA. The MI group from the indicated time point was compared to the SCAD group. GSEA plots showed the representative gene sets involved in inflammation, coagulation, metabolism and stress response. Gene sets with *p* value<0.05, *p*.adj < 0.25 and |NES| > 1 were considered to be significantly enriched. ES, enrichment score; NES, normalized enrichment score; OXPHOS, oxidative phosphorylation; *p*.adj, adjusted *p* value; *p*-val, *p* value; SCAD, stable coronary artery disease.

**Figure 7:**
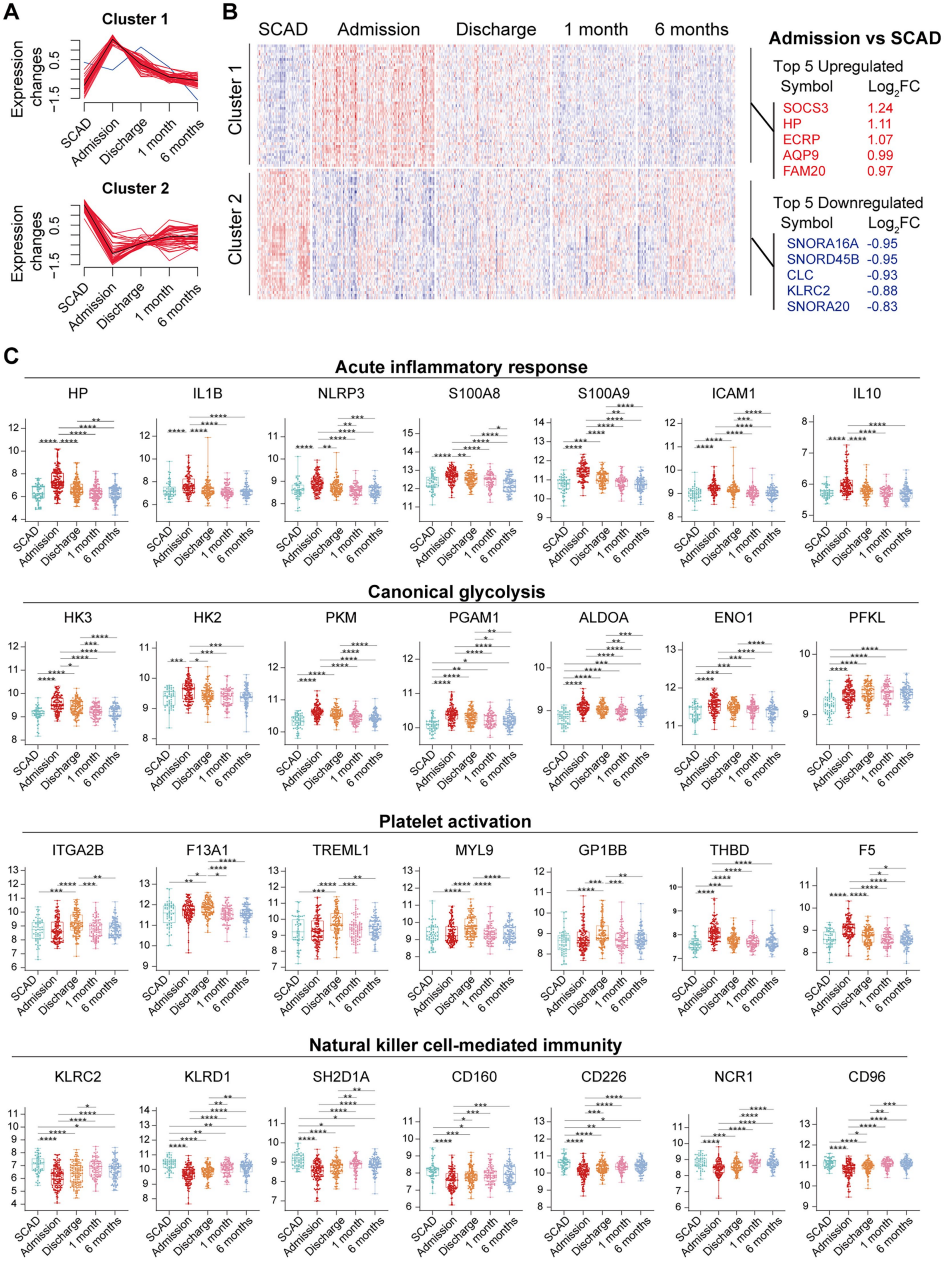
Temporal evolution of the post-MI PBMCs gene expression profiles assessed by Mfuzz. **(A)** Mfuzz soft clustering identified two clusters of DEGs. **(B)** Heatmap depicted the gene expression patterns of DEGs. Top 5 upregulated and downregulated DEGs at admission were specified. **(C)** The Box-Whisker plots showed the expression patterns of the representative leading-edge genes contributed most to the indicated enriched gene sets. The Box-Whisker plots denoted median + quartiles (box) and range (whiskers). Multiple-group comparisons were made using Kruskal–Wallis test with Dunn’s correction. *p < 0.05, **p < 0.01, ***p < 0.001, ****p < 0.0001. FC, fold change; SCAD, stable coronary artery disease.

We further integrated the data sets of GSE59867 (*n* = 436) and GSE62646 (*n* = 98) and incorporated all samples from four time points to investigate the temporal evolution of gene expression profiles of PBMCs from the MI patients. Dataset of GSE59867 contained a total of 436 PBMCs samples, including 46 samples from the SCAD patients, 111 samples from the MI patients at admission (on the 1st day of MI), 101 samples from the MI patients at discharge (after 4–6 days of MI), 95 samples from the patients 1 month after MI, and 83 samples from the patients 6 months after MI (22). The number of patients included decreased from one time point to the next because some patients failed to follow-up. Dataset of GSE62646 contained 98 PBMCs samples, including 14 samples from the SCAD patients, 28 samples from the MI patients at admission (on the 1st day of MI), 28 samples from the MI patients at discharge (after 4–6 days of MI) and 28 samples from the patients 6 months after MI (23). No patients from the original cohort of GSE62646 had been lost to follow-up. We merged the two datasets and normalized the matrix using *sva* package to control for variability between batches. The distribution of samples without (Figure 5A) and with (Figure 5B) batch-normalization adjustment was visualized by PCA plots. The normalized gene expression matrix was used for subsequent analyses. The combined dataset contained 534 PBMCs samples, including 60 samples from the SCAD patients, 139 samples from the MI patients at admission, 129 samples from the MI patients at discharge, 95 samples from the patients 1 month after MI and 111 samples from the patients 6 months after MI. GSVA score was computed per sample to quantify the pathway activities of each patient. Detailed GSVA results can be found in Supplementary Data Sheet 5. Next, we selected several hallmark pathways involved in inflammatory response and compared their GSVA score among different groups. As shown in Figure 5C, the GSVA score of inflammatory response and TNFA signaling *via* NFκB were increased at admission and returned to the baseline at discharge, while those of IL6/JAK/STAT3 signaling and glycolysis returned to the baseline 1 month after MI. The GSVA score of coagulation remained elevated until 1 month after MI, while GSVA score of OXPHOS was below the baseline level 6 months after MI. Next, the relative abundance of immune cells generated by xCell algorithm was compared among groups. As shown in Figure 5D, increased abundance of monocytes and neutrophils and decreased abundance of CD4^+^ and CD8^+^ T lymphocytes were observed in PBMCs from the MI patients at admission. The relative abundance of neutrophils returned to the baseline at discharge, while the level of monocytes seemed to be above the baseline even 6 months after MI. The relative abundance of B lymphocytes did not differ among groups. In addition, we visualized the dynamic expression patterns of the post-MI PBMCs using GSEA method. The MI groups from different time points were compared separately to the SCAD group. Detailed GSEA results can be found in Supplementary Data Sheet 6. As shown in Figure 6, the signals of most of the hallmark pathways related to inflammation and metabolic alterations peaked at admission and declined thereafter. In contrast, the signals of natural killer (NK) cell-mediated immunity were lower at admission and returned to the baseline over time. Interestingly, we noted a significant downregulation of OXPHOS in PBMCs 6 months after MI, which was consistent with the GSVA results shown in Figure 5C. Thus, both GSEA and GSVA algorithms confirmed that the signals of OXPHOS in PBMCs 6 months after MI was downregulated. To further characterize the temporal dynamics of the post-MI PBMCs, we used Mfuzz method to cluster the DEGs according to their expression patterns throughout the time course. A total of 94 DEGs with 1.5-fold enrichment (*p* value<0.05 and |log_2_FC| > 0.585) in at least one time point were taken into Mfuzz analysis. Two clusters of DEGs were identified by Mfuzz (Figures 7A,B). Cluster 1 contained 45 genes whereas cluster 2 contained 49 genes. Detailed Mfuzz results can be found in Supplementary Data Sheet 7. The expression level of genes in cluster 1 (e.g., SOCS3, HP, ECRP, APQ9, and FAM20) was increased on the first day of MI and decreased thereafter, while the expression in cluster 2 (e.g., SNORA16A, SNORD45B, CLC, KLRC2, and SONRA20) were decreased on the first day of MI and increased thereafter. The dynamic expression patterns of the representative leading-edge genes contributed most to the enriched GSEA terms were depicted using boxplots (Figure 7C). These results suggest a possibly dynamic expression pattern of genes involved in inflammatory response, metabolic reprogramming and platelet activation in PBMCs during a 6-month observation period.

In addition, as shown in Figure 6, the signals of platelet activation peaked at discharge rather than at admission. To our best knowledge, this observation has not been previously reported. To avoid error possibly caused by consolidation of datasets, GSEA analysis for the dataset GSE59867 and GSE62646 was performed separately. As shown in Supplementary Figures 1A,B, the signals of hallmark platelet activation pathways were higher at discharge than admission. It has been demonstrated that the number of the circulating reticulated platelets (RPs) is elevated in MI patients who have received PCI and anti-platelet therapies and remained relatively stable during the first few months after the onset (43). Given that RPs are newly-formed immature and hyperactive platelets containing significantly more RNA compared to mature platelets (MPs) (44), we further tested the possibility that RPs might contribute to higher signals of platelet activation. First, we compared the transcriptomic profiles of RPs and MPs (45). The results showed that multiple pathways were enriched in RPs, including coagulation, platelet activation, platelet aggregation and platelet degranulation (Supplementary Figure 1C). In particular, transcripts involved in platelet reactivity, clotting and cell–cell interactions (e.g., DMTN, SELP, ITGA2B, ITGB3, GP6, TREM1, TUBB1, and C6orf25) were upregulated in the RNA-rich RPs (Supplementary Figure 1D), indicating that RPs have a prothrombotic transcriptomic profile Moreover, the expression levels of these hallmark RP genes in MI PBMCs were higher at discharge than admission (Supplementary Figure 1E). Thus, these results suggest that higher platelet activation could be in part explained by possibly enhanced platelet turnover and increased RPs in MI patients at discharge.

### 3.4. HF PBMCs are characterized by elevated and prolonged expression of genes implicated in inflammation and coagulation

**Figure 8:**
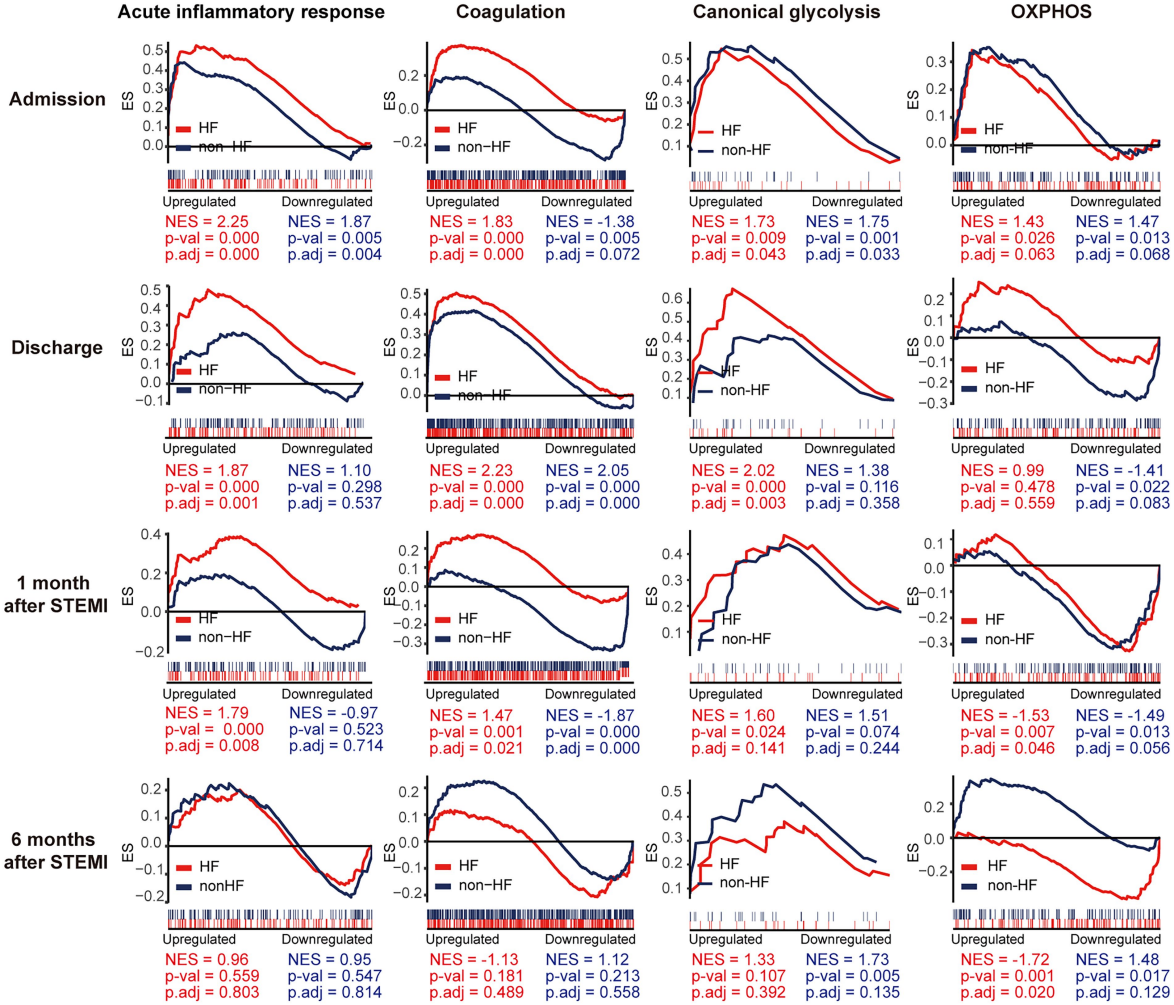
Temporal evolution of the post-MI PBMCs gene expression profiles in the patients with and without the long-term HF. The HF group and the non-HF group were compared separately to the SCAD group. The representative gene sets involved in acute inflammatory response, coagulation, canonical glycolysis and OXPHOS were presented. Gene sets with a *p* value<0.05, *p*.adj < 0.25 and |NES| > 1 were considered to be significantly enriched. ES, enrichment score; HF, heart failure; NES, normalized enrichment score; OXPHOS, oxidative phosphorylation; *p*.adj, adjusted *p* value; *p*-val, *p* value; SCAD, stable coronary artery disease; STEMI, ST-elevation myocardial infarction.

**Figure 9:**
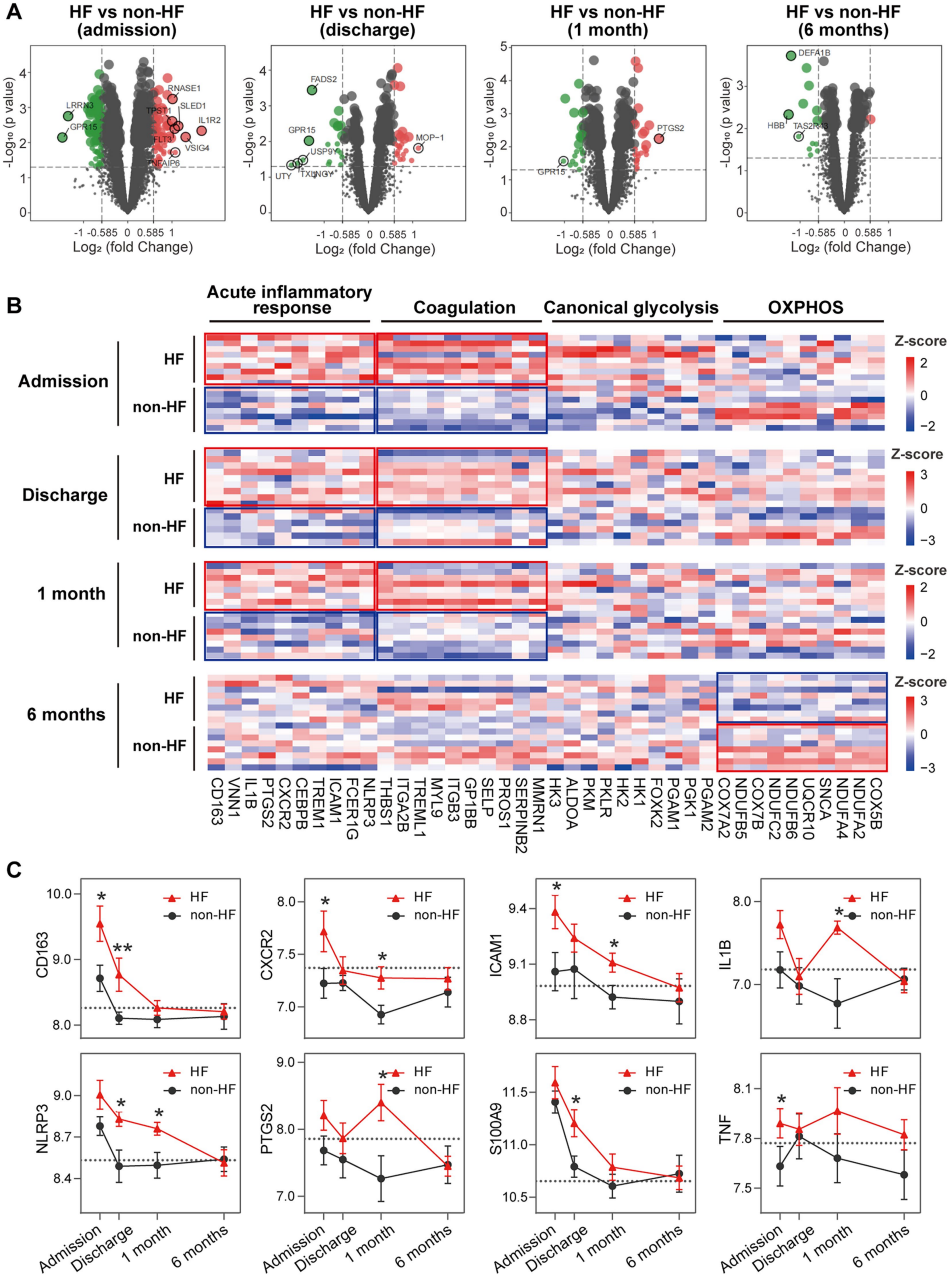
Elevated and prolonged expression of genes implicated in inflammation and coagulation in HF patients. **(A)** Volcano plots depicted the DEGs. DEGs with *p* < 0.05 and |log_2_FC| > 1 were circled and labeled. **(B)** Heatmaps depicted the expression of representative leading edge genes contributed most to the indicated gene sets. Statistical enrichment was highlighted with the solid-line boxes. **(C)** Line graphs showed the dynamic and heterogeneous expression patterns of hallmark inflammation-associated transcripts in the HF and non-HF groups. The dashed line represented the average gene expression level in the SCAD group. Data were expressed as mean ± SEM. Two-group comparisons at each time point were made using the Mann–Whitney non-parametric test with *p* value<0.05 defining statistical significance. *p < 0.05, **p < 0.01. HF, heart failure; OXPHOS, oxidative phosphorylation; SCAD, stable coronary artery disease.

To gain additional insights into the molecular signatures of PBMCs associated with the development of post-MI HF, we analyzed GSE59867 data set and extracted samples from the patients with or without clinical HF 6 months after MI. Samples from the SCAD patients (*n* = 46) in GSE59867 data set served as controls. According to the original study, the post-MI patients who volunteered for a visit 6 months after the onset were divided into the HF group (*n* = 9) and the non-HF group (*n* = 8) (22). Patients in the HF group were characterized by decreased LVEF% (39.3 ± 8.4%) and elevated level of NT-pro-BNP (918.3 ± 848.5 pg./ml) 6 months after the onset of MI. The sample size of the HF group at admission, at discharge, 1 month and 6 months after MI was 9, 9, 8, 8, respectively. The number of patients included may decreased from one timepoint to the next because of the missing data in the original study. Patients in the non-HF group did not develop HF 6 months after MI. The NT-pro-BNP levels and LVEF values in the non-HF group 6 months after the onset of MI were 62 ± 14.1 pg/ml and 66.8 ± 1.9%, respectively. The sample size of the HF group at admission, at discharge, 1 month and 6 months after MI was 8, 6, 8, 8, respectively. The number of patients at discharge decreased because of the missing data in the original study. The baseline demographic and clinical characteristics of the HF group and the non-HF group were showed in the original publication (22). There was no significant difference between the two groups in the following parameters: gender, age, BMI, hypertension, diabetes, hypercholesterolemia, previous MI, and smoking (22). All patients in the HF group and the non-HF group received standard medications, including aspirin, beta blockers, ACE inhibitors, statins, etc. Firstly, the HF group and non-HF group were analyzed against the SCAD group using GSEA method. Detailed GSEA results at various time points can be found in Supplementary Data Sheet 8. Based on the aforementioned results, acute inflammatory response, coagulation, canonical glycolysis and OXPHOS were chosen as representative gene sets. As shown in Figure 8, MI patients at admission were marked by a significant enrichment of genes in inflammatory response, coagulation, canonical glycolysis and OXPHOS in their PBMCs, irrespective of the development of HF. Gene sets of acute inflammatory response were consistently enriched until 1 month after MI in the HF group, whereas this enrichment was not detected at discharge in the non-HF group. Meanwhile, coagulation gene set was positively enriched in the HF group and negatively enriched in the non-HF group 1 month after MI. Next, we visually compared the difference in PBMCs gene expression profiles between the HF group and the non-HF group. Detailed GSEA results at various time points can be found in Supplementary Data Sheet 9. As shown in Figure 9A, compared to the non-HF group, the HF group had 142 DEGs at admission (61 upregulated and 81 downregulated), 42 DEGs at discharge (23 upregulated and 19 downregulated), 45 DEGs 1 month after MI (25 upregulated and 20 downregulated) and 14 DEGs 6 months after MI (1 upregulated and 13 downregulated). GSEA revealed that acute inflammatory response and coagulation gene sets were significantly enriched in the HF group at admission, at discharge and 1 month after MI (Table 2), indicating that post-MI development of HF is associated with elevated and prolonged inflammation and coagulation abnormalities. Furthermore, OXPHOS gene set was negatively enriched in the HF group 6 months after MI, suggesting potential mitochondrial dysfunction of PBMCs in the post-MI HF patients. The core genes contributed most to the enriched gene sets were further assessed by GSEA leading edge analysis. The results were visualized using a heatmap (Figure 9B). The temporal expression patterns of representative pro-inflammatory transcripts (e.g., CD163, CXCR2, ICAM1, IL1B, NLRP3, PTGS2, S100A9, and TNF) in the PBMCs from the post-MI HF and non-HF groups were depicted using line plots (Figure 9C). Compared to the non-HF group, all of these genes exhibited a trend of upregulation at the early time points in the HF group, although not all differences were statistically significant. No differences were observed between the two groups 6 months after MI. Taken together, these results suggest that PBMCs from the post-MI HF patients are characterized by excessive activation of inflammation and coagulation pathways, indicating that prolonged elevation in inflammatory response and coagulation in PBMCs may predict the development of post-MI HF.

**Table 2:** Summary of GSEA results for the indicated gene sets.

Gene sets	Admission			Discharge			1 month			6 months		
	NES	*p*-val	*p*.adj	NES	*p*-val	*p*.adj	NES	*p*-val	*p*.adj	NES	*p*-val	*p*.adj
Acute inflammatory response	1.95	0.000	0.000	1.71	0.001	0.012	1.87	0.000	0.002	−1.11	0.276	0.677
Coagulation	2.18	0.000	0.000	1.67	0.000	0.001	2.13	0.000	0.000	−1.36	0.006	0.130
Canonical glycolysis	0.91	0.603	0.748	1.53	0.039	0.197	0.90	0.617	0.841	−0.88	0.620	0.895
OXPHOS	−0.78	0.926	0.955	1.39	0.051	0.230	1.29	0.082	0.266	−2.16	0.000	0.000

NES, normalized enrichment score; OXPHOS, oxidative phosphorylation; *p*.adj, adjusted *p* value; *p*-val, *p* value.

### 3.5. PBMCs from MI and post-MI HF patients may undergo metabolic reprogramming

**Figure 10:**
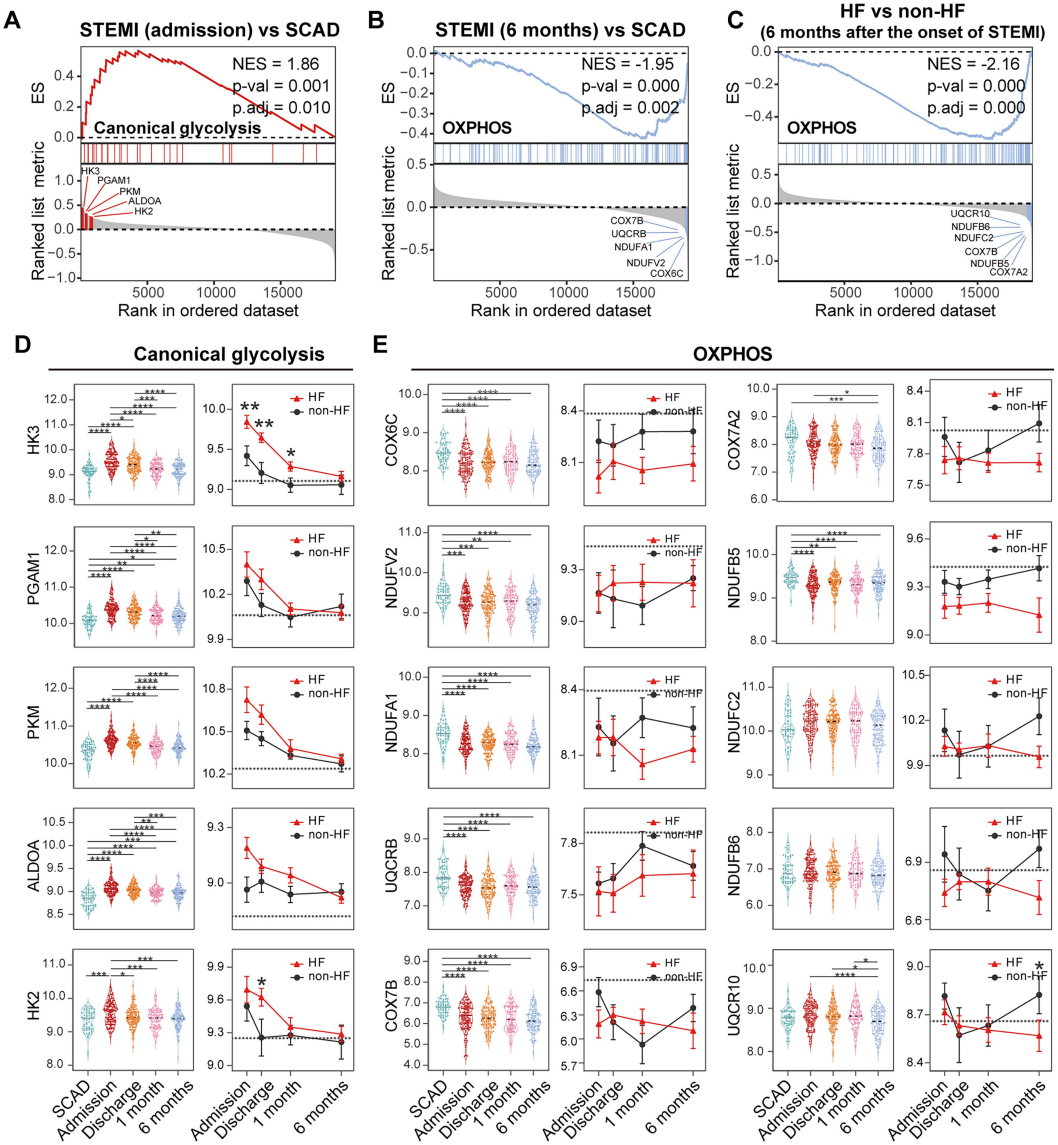
Metabolic reprogramming of PBMCs from the MI and post-MI HF patients. Comparison of the glycolysis and OXPHOS signals was made by GSEA between the MI group derived from the patients at admission and the SCAD group **(A)**, the MI group derived from the patients 6 months after MI and the SCAD group **(B)** and the HF group and the non-HF group from the patients 6 months after MI **(C)**. Gene sets with *p* value<0.05, *p*.adj < 0.25 and |NES| > 1 were considered to be significantly enriched. The top five leading edge genes contributed most to the indicated gene sets were specified on the plots. Boxplots (left panel) and line plots (right panel) showed the dynamic and heterogeneous expression patterns of glycolysis **(D)** and OXPHOS genes **(E)**. In the boxplots, multiple-group comparisons were made using Kruskal–Wallis test with Dunn’s correction. In the line plots, data were presented as mean ± SEM, two-group comparisons at each time point were made using the Mann–Whitney non-parametric test. The dashed line represented the average gene expression level in the SCAD group. For all tests applied, statistical significance was defined by *p* < 0.05. *p < 0.05, **p < 0.01, ***p < 0.001, ****p < 0.0001. ES, enrichment score; HF, heart failure; NES, normalized enrichment score; OXPHOS, oxidative phosphorylation; *p*.adj, adjusted *p* value; *p*-val, *p* value; SCAD, stable coronary artery disease; STEMI, ST-elevation myocardial infarction.

Metabolic reprogramming is mechanistically implicated in the inflammatory activation of immune cells (46). We thus further zoomed in on glycolysis and OXPHOS signals in PBMCs. GSEA revealed a significant upregulation of glycolysis and OXPHOS signals in PBMCs from the MI patients at admission (Figure 10A), consistent with the results shown in Figure 6. However, a significant downregulation of OXPHOS signals was noted in the patients 6 months after MI compared to the SCAD patients (Figure 10B), which was in agreement with the results shown in Figures 5B, 6. It is worth noting that similar observations were made in the HF patients 6 months after MI compared to those without HF (Figure 9C). The top five leading-edge genes contributed most to the canonical glycolysis and OXPHOS gene sets were marked on the GSEA plots (Figures 10A–C). The expression level of glycolytic genes such as HK3, PGAM1, PKM, ALDOA, and HK2 was increased at admission and decreased thereafter in both HF and non-HF groups (Figure 10D). Compared to the non-HF group, these glycolytic genes exhibited a trend of upregulation at the early time points in the HF group, although not all differences reached statistical significance. The expression level of OXPHOS genes such as COX6C, NDUFV2, NDUFA1, UQCRB, and COX7B was below the baseline after MI (Figure 10E). No significant differences in the expression level of these genes were noted between the HF group and non-HF group. However, although statistically insignificant, lower expression of OXPHOS genes COX7A2, NDUFB5, NDUFC2, NDUFB6, and UQCR10 was noted in the HF group 6 months after MI (Figure 10E). The statistical insignificance was possibly due to small sample sizes and the heterogeneity of the HF patients. Nevertheless, these results suggest a possibility that PBMCs from the post-MI HF patients are characterized by prolonged metabolic abnormalities.

### 3.6. Diversity and heterogeneity of PBMCs from the patients with chronic HF

**Figure 11:**
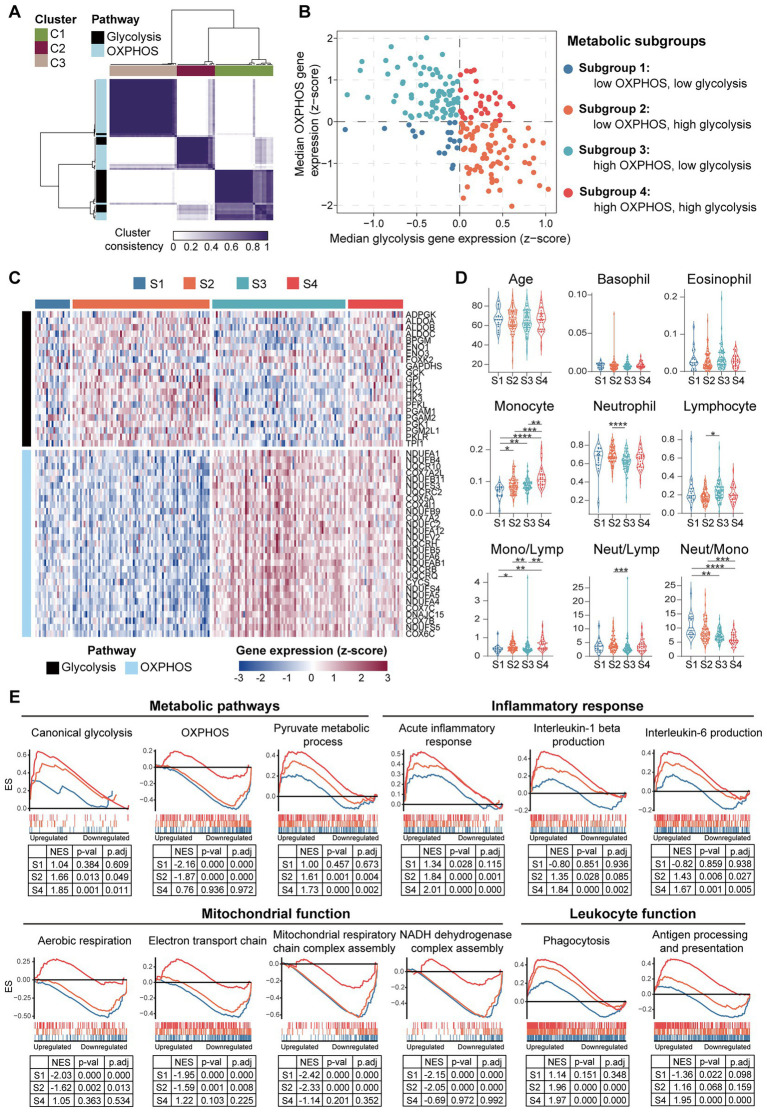
Diversity and heterogeneity of PBMCs from the HF patients. **(A)** A three-cluster solution of PBMCs yielded by consensus clustering (*n* = 197). **(B)** A scatter plot showed the sample distribution in the four quadrants defined by the relative median expression level of the co-expressed glycolysis and OXPHOS genes. **(C)** Heatmap depicted the relative expression level of the co-expressed glycolysis and OXPHOS genes across the indicated subgroups. **(D)** Violin plots showed the differences of the indicated parameters revealed by multiple-group comparisons using Kruskal–Wallis test with Dunn’s correction. **(E)** GSEA plots depicted the phenotypic heterogeneity of PBMCs from the HF patients. Gene sets with *p* value<0.05, *p*.adj < 0.25 and |NES| > 1 were considered to be significantly enriched. ES, enrichment score; Lymp, lymphocyte; Mono, monocyte; Neut, neutrophil; NES, normalized enrichment score; OXPHOS, oxidative phosphorylation; *p*.adj, adjusted *p* value; *p*-val, *p* value; S, subgroup.

To further clarify if the downregulation of OXPHOS genes in PBMCs is universally or selectively associated with the HF patients, and to determine whether metabolic reprogramming is correlated with inflammatory response in PBMCs from the HF patients, GSE77343 data set containing 197 PBMCs samples from chronic HF patients was analyzed. Consensus clustering was performed to stratify the HF patients based on their metabolic characteristics. After developing a custom gene set containing 26 glycolytic genes and 47 OXPHOS genes (Supplementary Data Sheet 1), a group of robustly co-expressed glycolytic (*n* = 21) and OXPHOS (*n* = 29) genes identified by consensus clustering was subjected to molecular subtyping (Figure 11A). Based on the median relative expression levels of the co-expressed glycolytic and OXPHOS genes, PBMCs from the HF patients were divided into four subgroups based on their metabolic profiles, namely, subgroup 1 (S1: low OXPHOS and low glycolysis), subgroup 2 (S2: low OXPHOS and high glycolysis), subgroup 3 (S3: high OXPHOS and low glycolysis), and subgroup 4 (S4: high OXPHOS and high glycolysis; Figure 11B). Heatmap was generated to depict the expression patterns of the glycolytic and OXPHOS genes (Figure 11C). Subgroup 2 defined the largest group of cases (75/197; 38.1%), followed by subgroup 3 (73/197; 37.1%), subgroup 4 (30/197; 15.2%), and subgroup 1 (19/197; 9.6%). Since it has been reported that under aerobic conditions, the energy metabolism of PBMCs primarily relies on mitochondrial OXPHOX rather than cytoplasmic glycolysis (47), the S3 subgroup most closely resembled the metabolic profiles of the normal PBMCs. Thus, further comparisons were made against the S3 subgroup. To answer whether the metabolic profiles of PBMCs was associated with inflammation, blood routine analysis and GSEA were carried out for each group. According to the results detailed in the original study, the S1 subgroup had a lower proportion of monocytes and higher neutrophil-to-monocyte ratio (NMR) than the S3 subgroup; the S2 subgroup had a higher proportion of neutrophils, lower proportion of lymphocytes, higher neutrophil-to-lymphocyte ratio (NLR) and higher monocyte-to-lymphocyte ratio (MLR) than the S3 subgroup; the S4 subgroup had a higher proportion of monocytes and higher MLR than the S3 subgroup (Figure 11D). Neither age, nor the proportions of basophils and eosinophils differed among the four subgroups based on their PBMCs metabolic profiles. GSEA was performed to further validate the heterogeneity of PBMCs in different subgroups. Detailed GSEA results can be found in Supplementary Data Sheet 10. As shown in Figure 11E, canonical glycolysis gene set was positively enriched in the S2 and S4 subgroups, whereas OXPHOS gene set was negatively enriched in the S1 and S2 subgroups. The S2 and S4 subgroups, which were characterized by a highly glycolytic phenotype, exhibited a positive enrichment of gene sets involved in inflammatory response and phagocytosis. However, the S1 and S2 subgroups, which displayed a low OXPHOS phenotype, displayed a negative enrichment of gene sets implicated in mitochondrial function. Thus, the results here demonstrate the diversity and heterogeneity of PBMCs from HF patients.

## 4. Discussion

The work here primarily reveals the temporal evolution of gene expression profiles in PBMCs and the heterogeneity of PBMCs in MI patients with or without long-term clinical outcome of HF. Moreover, the work here highlights the associations of inflammatory responses and metabolic reprogramming in PBMCs from the post-MI HF patients, which may help better understand the mechanisms of inflammation in the development of post-MI HF.

First, the work here suggests that PBMCs from the MI patients undergo extensive changes characterized by pro-inflammatory activation, metabolism remodeling and enhanced leukocyte-platelet interactions. A robust and ubiquitous upregulation of hallmark pro-inflammatory transcripts such as CLEC5A, HP, IL1B, NLRP3, S100A8, and S100A9 is detected in PBMCs from the MI patients (Figure 3F). Meanwhile, this pattern is noted in nearly all datasets analyzed in the present study and pertains to not only PBMCs but also peripheral whole blood, thrombus-derived leukocytes, CECs, platelets and exosomes (Figures 3F, 4D). Furthermore, the expression of genes encoding key rate-limiting glycolysis enzymes such as HK1, HK3, and PKM appears to be predominantly elevated in leukocytes but not in CECs, platelets and exosomes (Figure 3F). Inflammatory leukocytes could rewire their metabolism and become highly glycolytic under hypoxic and stress conditions (48, 49). It is thus likely that PBMCs swiftly undergo metabolic reprogramming and shift toward glycolysis in the inflammatory processes in the context of MI. Furthermore, our results suggest that LDGs could contribute to the enrichment of neutrophil-specific gene sets in PBMCs from the MI patients (Figures 3E,F). LDGs are present in PBMCs layer during separation due to low density and have been shown to play a pro-inflammatory role in cancer (50), COVID-19 (51), and autoimmune disorders (52). We also identify the upregulation of platelet-specific genes, for instance, GP1BB, GP1BA, ITGA2B, TREML1, in PBMCs from the MI patients (Figure 7C), which may in part be attributed to the formation of leukocyte-platelet aggregates (39, 40) or platelet-derived extracellular vesicles (PEV) (53). Enrichment of platelet-specific genes in PBMCs from the MI patients suggests the interaction of circulating leukocytes and platelets during MI. These findings highlight an increasingly appreciated role of inflammation as a fundamental feature of MI.

Secondly, our results suggest that the evolution of gene expression profiles of PBMCs from MI patients is possibly dynamic. From a pathological perspective, the post-MI cardiac repair process could be broadly divided into three overlapping phases: the inflammatory phase, the reparative/proliferative phase and the maturation phase (54, 55). The early post-MI inflammatory phase spans from day 0 to day 4 and is characterized by acute neutrophil and monocyte mobilization in the peripheral blood, as well as augmented infiltration of leukocytes in the infarcted tissue (55). Our results reveal that PBMCs from MI patients at admission display activation of pro-inflammatory signals, which attenuate over time and mostly return to the baseline level 1 month after MI, indicating the recovery of the circulating immune homeostasis after revascularization (Figures 5–7). While this is the prevailing view, data presented in this paper may further advance our understanding of inflammatory resolution by outlining a possibly dynamic transcriptomic framework of clinical PBMCs samples from MI patients during 6-months observation period. However, in light of the heterogeneity of the cellular composition of PBMCs, we could not rule out the possibility that one subset or specific subsets of immune cells (i.e., monocytes, neutrophils, T lymphocytes, or B lymphocytes) is highly dynamic, which drives the temporal changes of PBMCs observed in this work.

Thirdly, our results here suggest that the post-MI PBMCs is heterogeneous. In our analysis, 61.87% MI patients are classified into the hyper-inflammatory group, while the remaining patients (38.13%) display less-severe inflammation (Figure 2G). It is generally accepted that the severity of systemic inflammation is directly related to the extent of the post-MI myocardial damage (56). The stratification by consensus clustering suggests that MI patients at admission differ in the severity of inflammatory activation. Such discrepancy may be attributed to differences in ischemia time, infarct size, clinical signs of reperfusion, etc. Specifically, such heterogeneity is seen in the MI patients with and without the long-term outcome of HF (Figures 8, 9). Our results suggest that PBMCs collected from the post-MI HF patients have a distinct inflammatory and metabolic footprint compared with those from the post-MI non-HF patients (Figures 8, 9). Given the fact that such injury-induced inflammation could be a self-limited response for a considerable proportion of patients, this leads us to ask that whether anti-inflammatory treatments would be necessary for all MI patients or only for those with signs of excessive inflammation at the acute phase. Perhaps identifying patients at risk of developing HF for personalized treatment is a critical determinant for the success of anti-inflammatory strategy. This hypothesis is also consistent with the results from the CANTOS trial, which demonstrates that the monoclonal antibody targeting interleukin-1β canakinumab has clinical benefits in reducing adverse cardiovascular events in the MI patients with residual inflammatory risks (median hs-CRP level 4.2 mg/L at entry) (18). As for the previous MI patients or multivessel SCAD patients without overt inflammation (median hs-CRP level 1.6 mg/L at entry), anti-inflammatory methotrexate treatment is ineffective at reducing the cardiovascular events (57). Thus, the results here lend bioinformatic support to the future possibility that monitoring inflammatory indicators at multiple time points post MI may help better define clinical phenotypes and predict patient outcomes.

Additionally, our analyses here imply that platelet activation in MI PBMCs is higher at discharge than admission (Figure 6 and Supplementary Figures 1A). Moreover, our analyses show that the expression levels of hallmark RP genes in MI PBMCs are higher at discharge than admission (Supplementary Figure 1E). It has been reported that elevated circulating RPs are predictive of an insufficient response to the antiplatelet therapy (58) and have a prognostic value in patients with ACS (59). Our observations of platelet activation in MI PBMCs might be partially attributed to increased RPs in MI PBMCs, which requires further validation in future studies. In addition, these observations indirectly corroborate the association between prolonged or excessive activation of coagulation with the development of post-MI HF shown in Figure 8.

Lastly, the findings here raise the possibility that PBMCs are metabolically heterogeneous, which may contribute differently to the development of HF. Several studies have suggested that PBMCs are potentially informative in identifying systemic bioenergetic alterations (60, 61). It has been shown that systemic inflammation in the HF patients is associated with mitochondrial dysfunction in PBMCs. HF PBMCs are characterized by elevated expression of pro-inflammatory genes and reduced mitochondrial respiratory capacity (62). While this knowledge has advanced considerably over the past few years, our understanding of the metabolic processes within PBMCs in HF has been lagging behind. Cellular ATP mainly comes from OXPHOS and glycolysis. OXPHOS generates 36 ATP molecules from a single molecule of glucose, whereas glycolysis is less effective, generating two molecules of ATP from 1 molecule of glucose (63). However, boosting OXPHOS requires mitochondrial biogenesis, which is a complex and slow process. By contrast, glycolysis could be rapidly activated *via* the induction of glycolytic enzymes, which is a rapid process (63). Our initial analyses showed that the expression of OXPHOS genes in PBMCs from the post-MI HF patients is slightly yet insignificantly downregulated (Figures 10C,E). Given that HF is a multi-factorial disease with distinct clinical phenotypes (64), the small size of the cohort as well as the clinical heterogeneity of HF may account for the inconclusiveness of the foregoing results. We hypothesized that PBMCs form HF patients can be stratified into metabolic subgroups based on alterations in the expression of genes involved in OXPHOS and glycolysis. Therefore, we analyzed an independent cohort with a larger sample size to explore the metabolic changes in PBMCs from the HF patients. Our results reveal that HF PBMCs are heterogeneous with respect to glycolytic and OXPHOS gene expression profiles (Figures 11B,C). Meanwhile, defects in OXPHOS seem to be linked to abnormalities in mitochondrial function (S1 and S2 in Figure 11E), while enhanced glycolytic flux appears to be implicated in inflammatory response (S2 and S4 in Figure 11E). Although it is not yet clear whether this metabolic heterogeneity influences the clinical outcome, the subgroups with low level of OXPHOS gene expression (S1 and S2 subgroups in Figure 11D) have elevated NLR or NMR, both of which are associated with systemic inflammation and serve as independent risk factors for poor prognosis in the patients with SCAD (65), acute coronary syndrome (66), HF (67, 68), and COVID-19 (69). We thus propose that not all HF patients exhibit chronic systemic inflammation, emphasizing the necessity to stratify the HF patients by taking into account the molecular heterogeneity and diversity of PBMCs. These results also highlight a possibility that metabolic remodeling could be at play to support inflammation and constitutes a potential high-risk phenotype of HF.

Limitations of the present *in silico* analyses need to be considered. First, according to the original studies, the first sampling time is at hospital admission (on the 1st day of MI). In fact, “admission” is a vague term since the time from coronary occlusion to symptom presentation to the hospital admission varies among patients. This variability is expected to produce a biological variation in terms of the scale of acute phase response. However, this kind of disease state heterogeneity is almost inevitable in the clinical sample collection. From a clinical perspective, the subtype identification and risk stratification at specific time points (e.g., at admission, at discharge) may help guide stratified treatment in clinical practice. Second, our current analyses are based on the publicly available GEO database, unavailability of granular clinical information (e.g., age, comorbidities, specific treatments, and whole blood counts) limits our ability to perform in-depth analyses and come to more definitive conclusions. For instance, concomitant conditions and treatments (e.g., statins) may affect the inflammatory activation of PBMCs (70), which may cause heterogeneity of pro-inflammatory activation pathways in different MI patients. Meanwhile, our current analyses are based on bulk transcriptomic data generated from heterogenous PBMCs. With the advent of single cell sequencing technology (71), gene expression profiling and optimal interpretation of heterogenous cell populations such as PBMCs could be greatly improved. Future analyses are thus necessary utilizing the datasets generated from single cell sequencing platforms to further delineate changes associated with distinctly different cell subsets of PBMCs. Moreover, although the results from our analysis of immune classifications are in line with the previously published data (41, 72), providing another layer of evidence validating the phenotypes of MI PBMCs, this analysis is limited by using transcriptomic data (based on xCell deconvolution approach). Future prospective studies are needed to verify the preliminary conclusions derived from the current bioinformatic analyses. Lastly, although understanding the transcriptomic signatures from the clinically accessible PBMCs is the main focus of the current work, it is worth noting that the MI-associated inflammatory response is a complex and multifaced process that may involve pathophysiological alterations and interplay of the immune cells from different sources, for instance, circulating immune cells, residence immune cells in the heart as well as the immune cells from the surrounding pericardial adipose tissue (11–13). Therefore, additional analyses based on tissue-specific immune cells are also needed to gain a more comprehensive understanding of the MI-associated inflammatory response.

## 5. Conclusion

In summary, the major findings from this integrated whole-genome gene expression analysis highlight the notion that elevated inflammatory response is a marker of the poor prognosis of the MI patients. Meanwhile, our findings suggest the clinical heterogeneity and the crosstalk between inflammation and metabolic reprogramming in PBMCs in the context of MI and post-MI HF development. Thus, the work here provides new bioinformatic evidence supporting the implementation of timely and individualized anti-inflammatory treatments to prevent from the development of post-MI HF.

## Data availability statement

The original contributions presented in the study are included in the article/Supplementary material, further inquiries can be directed to the corresponding authors.

## Author contributions

TZ and YC supervised the study and critically revised the manuscript. YW performed the analyses and drafted the manuscript. All authors contributed to the article and approved the submitted version.

## Funding

This work was supported by the National Natural Science Foundation of China (82074049 to TZ).

## Conflict of interest

The authors declare that the research was conducted in the absence of any commercial or financial relationships that could be construed as a potential conflict of interest.

## Publisher’s note

All claims expressed in this article are solely those of the authors and do not necessarily represent those of their affiliated organizations, or those of the publisher, the editors and the reviewers. Any product that may be evaluated in this article, or claim that may be made by its manufacturer, is not guaranteed or endorsed by the publisher.

## Footnotes

^1^https://www.ncbi.nlm.nih.gov/geo/

^2^http://geneontology.org/

^3^https://www.genome.jp/kegg/

^4^https://xcell.ucsf.edu/

^5^https://metascape.org/gp/index.html
