# Supplementary material for: Integrated whole-genome gene expression analysis reveals an atlas of dynamic immune landscapes after myocardial infarction
Source: Front Cardiovasc Med. 2023 Mar 3;10:1087721. doi: 10.3389/fcvm.2023.1087721 (PMC10020602; doi:10.3389/fcvm.2023.1087721)
Supplement: Supplementary file 11 [file Data_Sheet_1.docx]

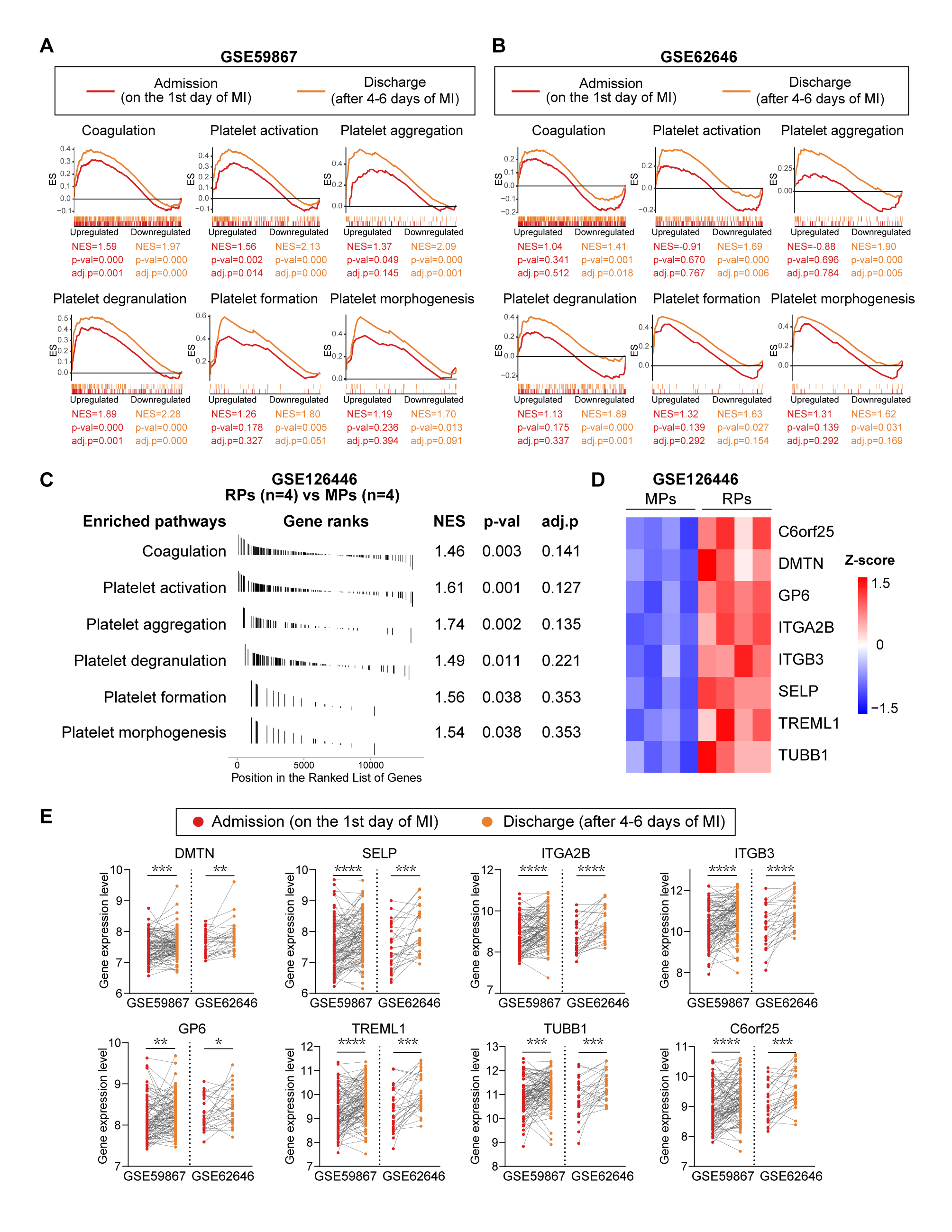
**Supplemental Figure 1. Elevated signals of platelet activation in PBMCs from MI patients on discharge.** GSEA plots showed a significant enrichment of gene sets involved in platelet activation in PBMCs from the MI patients on admission and on discharge, based on the results from data set of GSE59867 (**A**) and GSE62646 (**B**). Gene sets with p value<0.05, p.adj<0.25 and |NES|>1 were considered to be significantly enriched. **C**. Representative gene sets enriched in RPs. Each vertical bar represented one gene, with the location of the bar indicating the occurrence of that gene in the gene list and the height of the bar representing the relative fold change. Gene sets with p value<0.05, p.adj<0.25 and |NES|>1 were considered to be significantly enriched. **D**. Heatmap depicted the expression of hallmark RP genes in MPs and RPs. **E**. Significant differences in the expression level of hallmark RP genes in PBMCs were noted between the admission group and the discharge group. Statistical analysis was performed using a Wilcoxon matched-pairs test with p value<0.05 defining statistical significance. * p<0.05, ** p<0.01, *** p<0.001, **** p<0.0001. MPs, mature platelets; NES, normalized enrichment score; p.adj, adjusted p value; p-val, p value; RPs, reticulated platelets.
